# Special Issue “Drug Candidates for the Treatment of Infectious Diseases”

**DOI:** 10.3390/ph16091257

**Published:** 2023-09-06

**Authors:** Chung Man Chin, Jean Leandro Dos Santos

**Affiliations:** 1 School of Pharmaceutical Science, State University of São Paulo (Unesp), Araraquara 14800-903, Brazil; 2 Union of the Colleges of the Great Lakes (UNILAGO), School of Medicine, São José do Rio Preto 15030-070, Brazil

Infectious diseases encompass a range of conditions stemming from parasites [1,2], bacteria [3], viruses [4], fungi [5], or other parasitic organisms that negatively affect millions of individuals globally, particularly in low-income countries. Contemporary obstacles, such as the rise of resistance [6,7,8], the presence of severe adverse effects without adequate safety, subpar effectiveness, therapy non-adherence [9], and limited access to healthcare services, stand as barriers that must be surmounted. These challenges underscore the need to devise immediate and short-term strategies capable of reducing the burdens imposed by infectious diseases [1]. 

This Special Issue (SI) features the contribution of 20 papers and involves 161 authors representing 18 countries: Poland, Spain, France, Italy, Switzerland, Latvia, Mexico, Brazil, Ecuador, Japan, Korea, China, Russia, Kazakhstan, Israel, Saudi Arabia, Egypt, and Australia. All of the papers published in this SI investigated distinct microorganisms, such as protozoans (*Plasmodium falciparum*, *Trypanosoma cruzi*, *Giardia intestinalis*), nematode parasites (Haemonchus contortus, Nippostrongylus brasiliensis), viruses (SARS-CoV-2, hepatitis B virus and HIV), fungus (*Candida ssp*), mycobacteria (*Mycobacterium smegmatis*), bacteria (*Chromobacterium violaceum*, *Staphylococcus epidermidis*, *Enterococcus faecium*, *Staphylococcus aureus*, *Klebsiella pneumoniae*, *Acinetobacter baumannii*, *Pseudomonas aeruginosa*, *Enterobacter* spp., *Vibrio cholerae*) and the amoeba *Naegleria fowleri*.

This SI showcases several approaches for uncovering novel drugs derived from both synthetic and natural sources [10,11,12]. The compilation of papers delved into phenotypic assays [13], target-based drug discovery [14,15], high-throughput screening [16,17], computational methodologies [18], and natural biotechnological platforms [19,20,21]. These efforts aimed to pinpoint fresh prototypes warranting subsequent evaluation.

In the context of protozoans, Komatsuya and collaborators elucidated the impact of the natural product siccanin as an inhibitor of the mitochondrial electron transport chain (ETC) complex II of *P. falciparum* at nanomolar concentrations [22]. To delve into the structure–activity relationship (SAR) of gamhepathiopine, approximately 28 thieno[3,2-d]pyrimidines with substitutions at the 4-position were synthesized. These compounds were discovered to demonstrate in vitro efficacy against both the erythrocytic stage of *P. falciparum* and the hepatic stage of *P. berghei* [23]. Barbosa and collaborators detailed the synthesis, SAR exploration, and assessment of brussonol derivatives active against *P. falciparum*, including resistant strains [24]. Argüello-García reported on the giardicidal effects of the neo-clerodane type diterpene named linearolactone. This compound was postulated to interact with the aldose reductase homologue (GdAldRed) from *G. intestinalis* [25]. In a separate study, Imperador and colleagues conducted a systematic review to evaluate the effects of the natural products resveratrol and curcumin for Chagas disease [26].

Nematode infections are categorized among the neglected tropical diseases. Marchand and colleagues have devised a compelling in vitro screening platform that relies on fluorescence-based measurements to assess parasite viability [27]. Meanwhile, Shanley and collaborators conducted high-throughput screening employing the ‘pandemic response box’. Through this approach, they identified a prospective quinoline derivative exhibiting IC_50_ values of 3.4 μM and 7.1 μM against the motility of *H. contortus* larvae and adult *C. elegans* [28].

In the realm of viral infections, Assylbekova and colleagues were pioneers in reporting that camostat does not impede the proteolytic activity of neutrophil serine protease during SARS-CoV-2 infection [29]. Singh and Arkin detailed the impact of arapladib and flumatinib in obstructing the 3a ion channel linked to SARS-CoV-2 [30]. For HIV, Lopes and her research team elucidated the epigenetic modulation through histone deacetylase inhibitors. This approach is being explored in the ‘kick and kill’ strategy, with the aim of reactivating HIV from its reservoirs [31].

Spunde and colleagues undertook the synthesis and evaluation of capsid assembly modulators for hepatitis B virus (HBV), revealing a robust antiviral compound that demonstrated reduced cytotoxicity [32]. The acquisition and evaluation of homoisoflavonoid derivatives against Candida species exhibited a promising antifungal effect by diminishing ergosterol biosynthesis [33]. In a separate study, Buravchenko and collaborators synthesized 2-acyl-3-trifluoromethylquinoxaline 1,4-dioxides that displayed antibacterial effects against Gram-positive strains, as well as anti-mycobacterial activity [34].

The search for new antibacterial agents was also described by Mingoia et al. [35] and Frolov et al. [36], which described cinnamic acid-based and quaternary ammonium-based derivatives. Calzada et al. [37] detailed the effects of incomptines A and B against *Vibrio cholerae* and its enterotoxin. Utilizing computational chemistry, strategies were devised for the structure-based lead optimization of TcaR inhibitors [38], as well as the identification of alpha and beta-adrenoreceptor blockers targeting bacterial virulence factors [39]. Rizo-Liendo and collaborators elucidated the effects of naphthyridine derivatives in inducing programmed cell death in the pathogenic amoeba *Naegleria fowleri* [40].

This Special Issue delves into the core challenges associated with drug discovery for infectious diseases, showcasing these obstacles through a collection of exemplar cases outlined in the published articles. Finally, the Guest Editors would like to extend their gratitude for the collaborative spirit and diligent contributions of all authors in submitting their papers and reviewers for their valuable contributions. 

## References

[1] Baker R.E., Mahmud A.S., Miller I.F., Rajeev M., Rasambainarivo F., Rice B.L., Takahashi S., Tatem A.J., Wagner C.E., Wang L.F. (2022). Infectious disease in an era of global change. Nat. Rev. Microbiol..

[2] De Rycker M., Baragaña B., Duce S.L., Gilbert I.H. (2018). Challenges and recent progress in drug discovery for tropical diseases. Nature.

[3] Hutchings M.I., Truman A.W., Wilkinson B. (2019). Antibiotics: Past, present and future. Curr. Opin. Microbiol..

[4] Ma-Lauer Y., Lei J., Hilgenfeld R., von Brunn A. (2012). Virus-host interactomes—Antiviral drug discovery. Curr. Opin. Virol..

[5] Prescott T.A.K., Hill R., Mas-Claret E., Gaya E., Burns E. (2023). Fungal Drug Discovery for Chronic Disease: History, New Discoveries and New Approaches. Biomolecules.

[6] Lewis K. (2020). The Science of Antibiotic Discovery. Cell.

[7] Huemer M., Mairpady Shambat S., Brugger S.D., Zinkernagel A.S. (2020). Antibiotic resistance and persistence-Implications for human health and treatment perspectives. EMBO Rep..

[8] Darby E.M., Trampari E., Siasat P., Gaya M.S., Alav I., Webber M.A., Blair J.M.A. (2023). Molecular mechanisms of antibiotic resistance revisited. Nat. Rev. Microbiol..

[9] Alipanah N., Jarlsberg L., Miller C., Linh N.N., Falzon D., Jaramillo E., Nahid P. (2018). Adherence interventions and outcomes of tuberculosis treatment: A systematic review and meta-analysis of trials and observational studies. PLoS Med..

[10] Katz L., Baltz R.H. (2016). Natural product discovery: Past, present, and future. J. Ind. Microbiol. Biotechnol..

[11] Atanasov A.G., Zotchev S.B., Dirsch V.M., Supuran C.T., International Natural Product Sciences Taskforce (2021). Natural products in drug discovery: Advances and opportunities. Nat. Rev. Drug Discov..

[12] Blakemore D.C., Castro L., Churcher I., Rees D.C., Thomas A.W., Wilson D.M., Wood A. (2018). Organic synthesis provides opportunities to transform drug discovery. Nat. Chem..

[13] Herath H.M.P.D., Taki A.C., Rostami A., Jabbar A., Keiser J., Geary T.G., Gasser R.B. (2022). Whole-organism phenotypic screening methods used in early-phase anthelmintic drug discovery. Biotechnol. Adv..

[14] Malhotra S., Thomas S.E., Ochoa Montano B., Blundell T.L. (2016). Structure-guided, target-based drug discovery—Exploiting genome information from HIV to mycobacterial infections. Postepy Biochem..

[15] Gilbert I.H. (2013). Drug discovery for neglected diseases: Molecular target-based and phenotypic approaches. J. Med. Chem..

[16] Sykes M.L., Avery V.M. (2013). Approaches to protozoan drug discovery: Phenotypic screening. J. Med. Chem..

[17] Blay V., Tolani B., Ho S.P., Arkin M.R. (2020). High-Throughput Screening: Today’s biochemical and cell-based approaches. Drug Discov. Today.

[18] Ejalonibu M.A., Ogundare S.A., Elrashedy A.A., Ejalonibu M.A., Lawal M.M., Mhlongo N.N., Kumalo H.M. (2021). Drug Discovery for Mycobacterium tuberculosis Using Structure-Based Computer-Aided Drug Design Approach. Int. J. Mol. Sci..

[19] Boparai J.K., Sharma P.K. (2020). Mini Review on Antimicrobial Peptides, Sources, Mechanism and Recent Applications. Protein Pept. Lett..

[20] Wang C., Hong T., Cui P., Wang J., Xia J. (2021). Antimicrobial peptides towards clinical application: Delivery and formulation. Adv. Drug Deliv. Rev..

[21] De Cena G., Scavassa B., Conceição K. (2022). In Silico Prediction of Anti-Infective and Cell-Penetrating Peptides from Thalassophryne nattereri Natterin Toxins. Pharmaceuticals.

[22] Komatsuya K., Sakura T., Shiomi K., Ōmura S., Hikosaka K., Nozaki T., Kita K., Inaoka D. (2022). Siccanin Is a Dual-Target Inhibitor of Plasmodium falciparum Mitochondrial Complex II and Complex III. Pharmaceuticals.

[23] Lagardère P., Mustière R., Amanzougaghene N., Hutter S., Franetich J., Azas N., Vanelle P., Verhaeghe P., Primas N., Mazier D. (2022). 4-Substituted Thieno[3,2-d]pyrimidines as Dual-Stage Antiplasmodial Derivatives. Pharmaceuticals.

[24] Barbosa C., Ahmad A., Maluf S., Moura I., Souza G., Guerra G., Barros R., Gazarini M., Aguiar A., Burtoloso A. (2022). Synthesis, Structure&ndash;Activity Relationships, and Parasitological Profiling of Brussonol Derivatives as New *Plasmodium falciparum* Inhibitors. Pharmaceuticals.

[25] Argüello-García R., Calzada F., Chávez-Munguía B., Matus-Meza A., Bautista E., Barbosa E., Velazquez C., Hernández-Caballero M., Ordoñez-Razo R., Velázquez-Domínguez J. (2022). Linearolactone Induces Necrotic-like Death in Giardia intestinalis Trophozoites: Prediction of a Likely Target. Pharmaceuticals.

[26] Imperador C., Scarim C., Bosquesi P., Lopes J., Cardinalli Neto A., Giarolla J., Ferreira E., dos Santos J., Chin C. (2022). Resveratrol and Curcumin for Chagas Disease Treatment&mdash;A Systematic Review. Pharmaceuticals.

[27] Marchand A., Van Bree J., Taki A., Moyat M., Turcatti G., Chambon M., Smith A., Doolan R., Gasser R., Harris N. (2022). Novel High-Throughput Fluorescence-Based Assay for the Identification of Nematocidal Compounds That Target the Blood-Feeding Pathway. Pharmaceuticals.

[28] Shanley H., Taki A., Byrne J., Jabbar A., Wells T., Samby K., Boag P., Nguyen N., Sleebs B., Gasser R. (2022). A High-Throughput Phenotypic Screen of the ‘Pandemic Response Box’ Identifies a Quinoline Derivative with Significant Anthelmintic Activity. Pharmaceuticals.

[29] Assylbekova A., Zhanapiya A., Grzywa R., Sienczyk M., Schönbach C., Burster T. (2022). Camostat Does Not Inhibit the Proteolytic Activity of Neutrophil Serine Proteases. Pharmaceuticals.

[30] Singh A., Arkin I. (2022). Targeting Viral Ion Channels: A Promising Strategy to Curb SARS-CoV-2. Pharmaceuticals.

[31] Lopes J., Prokopczyk I., Gerlack M., Man Chin C., Santos J. (2021). Design and Synthesis of Hybrid Compounds as Epigenetic Modifiers. Pharmaceuticals.

[32] Spunde K., Vigante B., Dubova U., Sipola A., Timofejeva I., Zajakina A., Jansons J., Plotniece A., Pajuste K., Sobolev A. (2022). Design and Synthesis of Hepatitis B Virus (HBV) Capsid Assembly Modulators and Evaluation of Their Activity in Mammalian Cell Model. Pharmaceuticals.

[33] Ferreira A., Alves D., de Castro R., Perez-Castillo Y., de Sousa D. (2022). Synthesis of Coumarin and Homoisoflavonoid Derivatives and Analogs: The Search for New Antifungal Agents. Pharmaceuticals.

[34] Buravchenko G., Maslov D., Alam M., Grammatikova N., Frolova S., Vatlin A., Tian X., Ivanov I., Bekker O., Kryakvin M. (2022). Synthesis and Characterization of Novel 2-Acyl-3-trifluoromethylquinoxaline 1,4-Dioxides as Potential Antimicrobial Agents. Pharmaceuticals.

[35] Mingoia M., Conte C., Di Rienzo A., Dimmito M., Marinucci L., Magi G., Turkez H., Cufaro M., Del Boccio P., Di Stefano A. (2022). Synthesis and Biological Evaluation of Novel Cinnamic Acid-Based Antimicrobials. Pharmaceuticals.

[36] Frolov N., Detusheva E., Fursova N., Ostashevskaya I., Vereshchagin A. (2022). Microbiological Evaluation of Novel Bis-Quaternary Ammonium Compounds: Clinical Strains, Biofilms, and Resistance Study. Pharmaceuticals.

[37] Calzada F., Bautista E., Hidalgo-Figueroa S., García-Hernández N., Velázquez C., Barbosa E., Valdes M., Solares-Pascasio J. (2022). Understanding the Anti-Diarrhoeal Properties of Incomptines A and B: Antibacterial Activity against Vibrio cholerae and Its Enterotoxin Inhibition. Pharmaceuticals.

[38] Vuppala S., Kim J., Joo B., Choi J., Jang J. (2022). A Combination of Pharmacophore-Based Virtual Screening, Structure-Based Lead Optimization, and DFT Study for the Identification of S. epidermidis TcaR Inhibitors. Pharmaceuticals.

[39] Almalki A., Ibrahim T., Elhady S., Hegazy W., Darwish K. (2022). Computational and Biological Evaluation of alpha;beta;-Adrenoreceptor Blockers as Promising Bacterial Anti-Virulence Agents. Pharmaceuticals.

[40] Rizo-Liendo A., Arberas-Jiménez I., Martin-Encinas E., Sifaoui I., Reyes-Batlle M., Chao-Pellicer J., Alonso C., Palacios F., Piñero J., Lorenzo-Morales J. (2021). Naphthyridine Derivatives Induce Programmed Cell Death in Naegleria fowleri. Pharmaceuticals.

